# Prediction of Bone Metastasis in Breast Cancer Based on Minimal Driver Gene Set in Gene Dependency Network

**DOI:** 10.3390/genes10060466

**Published:** 2019-06-17

**Authors:** Jia-Nuo Li, Rui Zhong, Xiong-Hui Zhou

**Affiliations:** Hubei Key Laboratory of Agricultural Bioinformatics, College of Informatics, Huazhong Agricultural University, Wuhan 430070, China; ljn2126212@163.com (J.-N.L.); zr295110971@163.com (R.Z.)

**Keywords:** breast cancer, bone metastasis, gene dependency network, structure controllability theory, driver gene set

## Abstract

Bone is the most frequent organ for breast cancer metastasis, and thus it is essential to predict the bone metastasis of breast cancer. In our work, we constructed a gene dependency network based on the hypothesis that the relation between one gene and the risk of bone metastasis might be affected by another gene. Then, based on the structure controllability theory, we mined the driver gene set which can control the whole network in the gene dependency network, and the signature genes were selected from them. Survival analysis showed that the signature could distinguish the bone metastasis risks of cancer patients in the test data set and independent data set. Besides, we used the signature genes to construct a centroid classifier. The results showed that our method is effective and performed better than published methods.

## 1. Introduction

Breast cancer, one of the most frequent tumors, seriously affects human health [1,2]. Metastasis is the main cause of death in breast cancer patients, and bone is the most frequently metastatic organ [3]. Therefore, it is necessary to explore the mechanism of bone metastasis in breast cancer [4], and the research predicting bone metastasis in breast cancer is crucial [5].

At present, several works have attempted to explain the mechanism of bone metastasis in breast cancer by using molecular biology methods [6,7,8]. However, only a few studies have mined the prognostic makers and predicted the bone metastasis in breast cancer. Previously, we identified the key genes of breast cancer by identifying dysregulated pathways in the bone metastasis of breast cancer. The prediction model that we constructed to predict the risk of bone metastasis of breast cancer—the dysregulated pathway-based prediction model (DPBM) [9]—achieved a certain predictive effect, but the performance is still unsatisfactory.

Since cancer is a complex disease, a biological network that can reveal the gene regulatory relations in cancer becomes an effective tool [10]. In our previous work, we proposed a method to infer the gene dependency network which could uncover the gene regulatory relations during cancer metastasis, based on the hypothesis that the influence of one gene on the prognosis may depend on the other genes [11], and this network can be used to mine essential genes in the prognosis of cancer. Therefore, it may be promising to construct a gene dependency network in the bone metastasis of breast cancer.

In recent years, some exciting developments about the control of biological systems have been made [12], such as the structural controllability theory, referring to a system which could be controlled from one state to another by controlling several nodes (i.e., genes) in the system (biological network) [13]. Therefore, it is to use the gene dependency network in the bone metastasis of breast cancer as the biological system and identify the driver gene set based on the structural controllable theory. In addition, the driver gene set may be helpful for the construction of a prediction model.

In this work, we integrated the gene expression profiles and clinical information of 855 breast cancer patients to construct the gene dependency network, which could uncover the gene dependency relations in the process of bone metastasis in breast cancer [14]. Then, combined with the structural controllability theory we mined the driver nodes in the network as candidate features, based on which the gene signature was selected and a centroid classifier was constructed. We also validated our method by comparing it with other published methods.

## 2. Materials and Methods

### 2.1. Data Sets and Pre-Processing

We downloaded the gene expression profiles of breast cancer patients along with the clinical information from the UNC microarray database [15]. The downloaded data consisted of four microarray data sets: GSE*2034* [16], GSE*2603* [17], GSE*12276* [18], and NKI*295* [19]. The raw data was processed and the clinical information of the patients (time of bone-metastasis and status of bone metastasis) was extracted as in the previous work [15], and statistical “batch-effect correction” procedures were already performed on the data. For the sake of comparing the results with other methods, we adopted the same strategy as the DPBM to divide the data set into training, test, and independent data sets. GSE*2034* was used as the independent data set. In the remaining samples, *380* samples were selected as the training set and the other *189* samples were used as the test data set. For constructing the gene dependency network, the expression levels of all the genes and the clinical information were binarized. For a gene, the expression level of a patient was set as *0* if it was not more than the median of the gene’s expression levels in all the patients. Otherwise, it was set as *1*. As to the clinical information, in usual work, the 5-years rule is extensively recognized as the limitation in prognosis or metastasis risk prediction in breast cancer [20,21], and bone is the most common metastatic organ of breast cancer which occupied a large number of samples, so we chose 5 years as the threshold to determine the risk of bone metastasis of breast cancer patients. If a patient had bone metastasis within *5* years, they were set as high-risk (*1*); if they had bone-metastasis-free survival for more than *5* years, they were set in the low-risk group (*0*); otherwise, the patient was abandoned.

Furthermore, the human protein–protein interactions from HIPPIE (Human Integrated Protein–Protein Interaction rEference) [22] were also downloaded.

### 2.2. Construction of Gene Dependency Network

In our previous work, we proposed a method to infer the gene dependency network which could uncover the gene regulatory relations during cancer metastasis [11], based on the hypothesis that the influence of one gene on the metastasis may depend on another gene. Here, we applied it to infer the gene dependency network in the process of bone metastasis of breast cancer. Specifically, for two genes *A* and *B*, if the influence of gene *A* on phenotype (bone metastasis risk) is modulated by gene *B*, and we believe that gene *A* depends on gene *B*. Conditional mutual information (CMI) was applied to calculate the gene dependency relation between gene *A* and gene *B*, and a permutation test was performed to evaluate the significance of the gene dependency relation. The pipeline to construct the gene dependency network is shown as follows:

(1) In order to reduce the false discovery rate, only the gene pairs involved in the protein–protein interaction network were used as candidates. Here, all the gene pairs in HIPPIE [22] were set as candidates for gene dependency pairs.
(2) For each candidate gene dependency pair (e.g., gene *A* and gene *B*), we set the expression levels of gene *A* and gene *B* and the bone-metastasis risks of all the patients as a triple. The triple was sorted according to the expression level of gene *B* in ascending order. Then, the conditional mutual information was calculated by using Equation (1):
  (1) $$CMI(GeneA;risk|GeneB)=I_{high}\left(GeneA,risk\right)-I_{low}\left(GeneA,risk\right)$$
  where $I_{high}\left(GeneA,risk\right)$ is the mutual information of gene *A* and the bone-metastasis risks of *35*% cancer patients whose expression levels of gene *B* are high. $I_{low}\left(GeneA,risk\right)$ is the mutual information of gene *A* and the bone-metastasis risks of *35*% of cancer patients whose expression levels of gene *B* are low.
(3) A permutation test was proposed to calculate the *p*-value of the conditional mutual information for each gene pair. First, we randomly permuted the expression levels of gene *B*. Secondly, a random CMI was calculated for gene *A* and gene *B* using the method described in step (2). Thirdly, the random permutation was repeated *1000* times and *1000* random CMIs were obtained, and the *p*-value of the CMI was calculated based on the *1000* random CMIs. Finally, all the significant gene dependency pairs (*p*-value < *0.05*) were combined as the gene dependency network.

### 2.3. Structural Controllability of Networks

According to the controllability of complex networks [13], the non-linear processes (and linear processes) can be studied using the canonical linear and time-invariant dynamics.

The system described by Equation (2):

(2) $$\frac{dx\left(t\right)}{dt}=Ax\left(t\right)+Bu\left(t\right)$$

is controllable if it can be driven from any initial state to any desired final state in finite time, which is possible if and only if the controllability matrix *N* *× NM* has full rank. That is, the matrix *C* in Equation (3) follows Equation (4).

(3) $$C=\left(B,\mathrm{AB},A^{2}B,\ldots ,A^{N-1}B\right)$$

(4) rank(C)=N

Full controllability can be also posed by identifying the minimum number of driver nodes such that Equation (4) is satisfied [13]. However, it is a computationally prohibitive task for large networks [15], so it is to apply in practical problems. If it is possible to satisfy Equation (4) by selecting non-zero weights in *A* and *B*, the system is structurally controllable [23]. As the calculation process of structural controllability does not need to measure the link weights, it is acceptable in computational complexity. Furthermore, structural controllability can be solved by identifying the maximum matching in directed networks. Therefore, in this work, we applied the structure controllability theory to study the controllability of the bone metastasis system in breast cancer and identify the driver nodes in the system (direct network) as biomarker candidates.

### 2.4. Selecting the Driver Gene Sets by Controllability

Based on the hypothesis that the gene dependency network in the bone metastasis of breast cancer can reveal the biological mechanism in bone metastasis, the driver nodes that could structurally control the network may be crucial in the biological process of bone metastasis. Therefore, we identified these driver nodes as biomarker candidates.

As we described above, structural controllability can be solved by identifying the maximum matching in directed networks—that is, the minimal driver set that can drive all the nodes in the network. Here, the maximum matching algorithm [24] was applied to identify the minimal driver set in the gene dependency network, and these nodes were set as the driver nodes. However, as we know, the solutions of maximum matching in directed networks are not unique, especially in complex networks. In this work, we randomly permuted the index of nodes (keeping the dependency relations among the nodes) and ran this program for 500 times to get the frequencies of the occurrence of genes in the minimal driver set, and we chose the genes with the frequency of 500 as the candidate genes. That is, the nodes (genes) in all the driver sets were set as feature (biomarker) candidates.

In order to construct the classifier to predict the bone metastasis of cancer patients, the features should be different in the two patient groups (i.e., high-risk and low-risk groups). Therefore, the feature candidates were further selected by the *t*-test, so as to select the genes which were differentially expressed in the two groups (in the training data set). As a result, the driver nodes with *p*-values less than 0.002 were set as features.

### 2.5. Construction of the Classifier

In this work, we applied the centroid classifier to construct the prediction model. The first reason we chose it is that the centroid classifier is suitable for microarray data, which has the character of large feature size and few samples [25]. Secondly, the centroid classifier does not need to offer the extra adjustment of the parameter. Besides, the centroid classifier is difficult to overfit [26]. Supposing the number of bone metastases in breast cancer patients is *N*, and *s_1_,s_n_*, each *s_i_* represents the gene expression level vector from a patient. *s_i_* contains the *M* dimensions of the measurement value of the patient. Making a short explain about the *s_i_(i,j),* it represents the expression level of the *i*-th feature (gene) in the *j*-th patient. Above that, we denoted the *n^+^* patients belonging to the positive class (with the label 1) and the *n^-^* patients belonging to negative class (with the label 0) with Equation (5):

(5) $$N=n^{+}+n^{-}$$

Then, we could calculate the gene expression level centroid (mean value) vector with *M* dimensions separated by the class according to Equations (6) and (7) as follows:

(6) $${\vec{C}}^{+}=\frac{1}{n^{+}}\underset{S_{j}\in Class+}{\sum }S_{j}\left(i,j\right)$$

(7) $${\vec{C}}^{-}=\frac{1}{n^{-}}\underset{S_{j}\in Class-}{\sum }S_{j}\left(i,j\right)$$

In Equation (8)

(8) $$\vec{C}=\left({\vec{C}}^{+}+\;{\vec{C}}^{-}\right)/\;2$$

$\vec{C}$ means the mean vector of the centroids, and in Equation (9)

(9) $$\vec{w}={\vec{C}}^{+}\;-\;{\vec{C}}^{-}$$

$\vec{w}$ is the weight vector of the *M* features. If the feature vector of a sample is *s = (d_1_, …, d_M_)*, we can appraise the samples by the following Equation (10):

(10) $$\gamma =\langle \tilde{s}=-\vec{C},\vec{w}\rangle$$

If γ > 0, then $\tilde{s}$ is assigned to a positive class; otherwise, it is assigned to a negative class.

### 2.6. Tools and Package

The functions of the selected driver genes were annotated by GSEA (Gene Set Enrichment Analysis) [27]. The network was analyzed and visualized by using Cytoscape 3.6.1 and the survival analysis was performed using the R package “survival”.

## 3. Results

### 3.1. Basic Information of the Gene Dependency Network

We constructed the gene dependency network in the process of bone metastasis in breast cancer using conditional mutual information. It contained 10,163 gene pairs (Table S1) among 5380 genes (Figure S1). As a result, both in-degree and out-degree followed to the power law distribution, with the R-square values 0.946 and 0.942, and the correlation coefficients were 0.942 and 0.985. The average number of neighbors, both in-degree and out-degree, were 1.889. The results suggest that our gene dependency network is scale free and coincident with the typical characteristics of biological network.

In addition, we also found some remarkable pairs in the gene dependency network related to breast cancer and metastasis. For instance, MYC deregulation is conductive to development and progression in breast cancer [28]. Wnt signaling in breast cancer is important [29]. It has been reported that MYC can activate WNT in breast cancer [30], and in our work, Wnt1 was significantly dependent on MYC (with *p*-value of 0.002). As we know, TP53 is a famous gene in breast cancer which can influence cancer prognosis [31]. Migration in breast cancer can be suppressed by targeting MYO10 [32]. In our gene dependency network, TP53 and MYO10 had a significant dependency relation (with *p*-value of 0.013), and according to the literature, the expression of MYO10 is relevant to the expression of TP53 in breast tumors [33]. Furthermore, the BRCA1 gene is also crucial in breast cancer, which has an impact on breast cancer risk [34]. EZH2 is a marker of aggressive breast cancer [35]. It has been found that the downregulation of EZH2 decreases invasive breast cancer and requires BRCA1 [36]. In our network, EZH2 was dependent on BRCA1 with a *p*-value of 0.009. Therefore, our gene dependency network was reliable and could be used to study the bone metastasis of breast cancer.

### 3.2. Functional Annotation of the Driver Nodes

As we described above, structural controllability can be solved by identifying the maximum matching in directed networks, and the minimal driver set driving all the nodes in our gene dependency network may be important in the bone-metastasis process of breast cancer. Here, we selected driver nodes as feature candidates to construct the prediction model. The number of nodes in the minimal driver set in our network was 2483, which is about 46% of all the nodes. In a previous work [13], it was proved that about 80% of the nodes in biological networks should be controlled to satisfy the structural controllability, which may indicate that the gene dependency is easier to control.

Although the driver nodes in the network may be essential, there are two problems here hindering the application of the minimal driver gene set in feature selection. First of all, the size of the driver set is too large. As described above, almost 80% of all the nodes in biological networks are driver nodes. Even in our gene dependency network, 2483 genes were driver nodes. In addition, the solutions of the maximum matching are not unique, especially in complex networks. To solve these problems, we randomly permuted the index of nodes (keeping the dependency relations among the nodes) and ran this program for 500 times to get the frequency of the occurrence of genes in the minimal driver set, and we chose the genes with the frequency of 500 as the candidate genes. As a result, 1864 genes from the gene dependency network were selected as feature candidates (Table S2).

We also used GSEA [27] to investigate the function of the feature candidates. The top 10 KEGG (Kyoto Encyclopedia of Genes and Genomes) pathways are listed in Table 1.

The MAPK signaling pathway is the most significant pathway in breast cancer metastasis. Meanwhile, through suppression of the MAPK signaling pathway, metastasis of breast cancer cells can be reduced [37]. In addition, it has been reported that HGFK1 could inhibit bone metastasis in breast cancer through the TAK1/p38 MAPK signaling pathway [38]. Furthermore, the feature candidates are significantly enriched by pathways in cancer, with a *p*-value of 2.82 × 10^-13^. The feature candidates were significantly involved in the pathway of focal adhesion (*p*-value = 4.05 × 10^-11^), and it has been reported that focal adhesion kinase is a prominent determinant in the metastasis of breast cancer [39], has an effect on bone tumors, and may be crucial in bone metastasis [40]. The cytokine–cytokine receptor interaction is also significant (*p*-value = 8.22 × 10^-11^), and cytokines are relevant to the microenvironment of bone metastasis in breast cancer [41].

In conclusion, the genes which are frequently involved in the minimal driver gene sets are related to the metastasis, even bone metastasis in breast cancer.

### 3.3. The Selected Features

Based on the feature candidates (the genes involved in all the minimal driver gene sets), we selected 51 signature genes (Table 2) with *p*-values (*t*-test) less than 0.002 in order to find the differential expression genes.

In the signature, almost all the genes were related to cancer, and many genes were associated with bone metastasis of breast cancer. FSTL1 promotes bone metastasis by causing immune dysfunction [42]. It has been reported that Wnt signaling is related to bone metastasis in breast cancer [43] and that DIXDC1 can promote the metastasis of cancer cells by activating the Wnt signaling pathway [44]. Breast cancer patients with bone metastasis presented with reduced expression of NDRG1 and their survival was also influenced by NDRG1 expression [45]. Experiments have shown that a group of genes including MLPH can predict bone metastasis in breast cancer well [46]. CRIM1 is a potential risk factor of cancer that regulates the migration of cancer cells [47]. RANBP10 may help inhibit the growth of breast cancer cells [48]. The expression of RXRA also plays a key role in breast cancer [49].

In all, the genes in the signature were involved the minimal driver gene set, and they were distinguished in high-risk and low-risk groups. In addition, most of these genes are indeed related to the metastasis in breast cancer. Therefore, our gene signatures may be good features for classification in the bone metastasis of breast cancer.

### 3.4. Survival Analysis of Breast Cancer Patients

In order to predict the risk of bone metastasis in breast cancer patients, we divided the patients in the training data set into two groups (i.e., high-risk and low-risk groups). Using the genes in the signature as features, we constructed a centroid classifier to predict the bone metastasis of cancer patients based on the training data set. Then, the classifier was applied in the test data set and independent data set. We performed survival analysis of the patients in both the test data set and independent data set respectively to validate our classifier.

**Figure 1:**
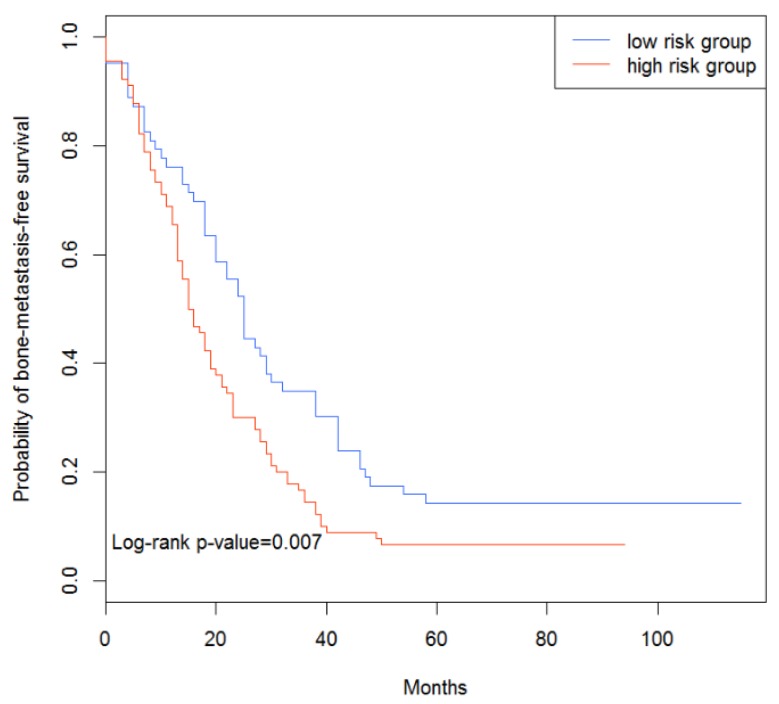
Survival analysis of test data set.

In the test data set, 71 patients were predicted as high-risk patients, and 98 patients were predicted as low-risk patients. Survival analysis showed that the bone-metastasis risks of the patients in the two groups were indeed different, with a *p*-value of 0.007 (Figure 1).

**Figure 2:**
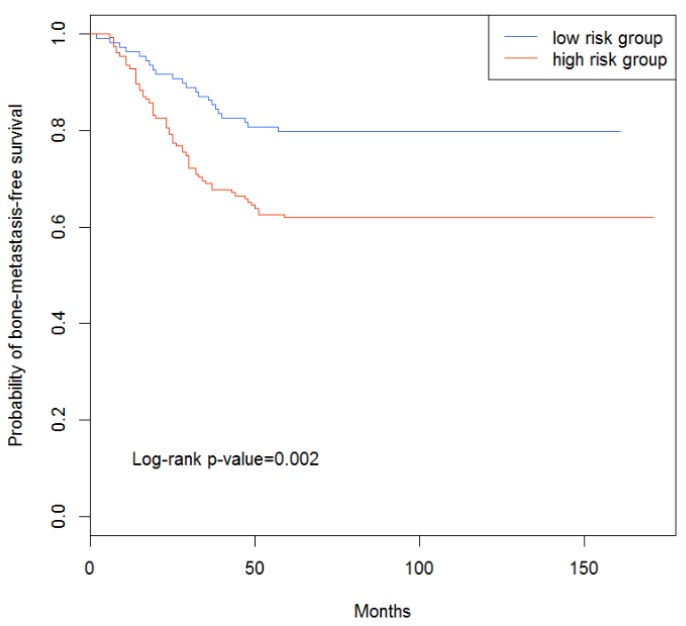
Survival analysis of the independent data set.

Among the 286 patients in the independent test set, 117 were classified as high-risk patients, and 169 patients were classified as low-risk patients. The *p*-value of the log-rank test of the patients in the two groups was 0.002 (Figure 2), indicating that the low-risk patients predicted by our method had longer survival time (bone-metastasis-free survival) than the high-risk ones.

From the results, it could be demonstrated that our classifier could distinguish the bone-metastasis risks in the test and independent data sets, indicating that the features (i.e., genes) we selected could indeed play a key role in the bone metastasis of breast cancer.

### 3.5. Comparison with Other Methods

In the previous work, the dysregulated pathway-based prediction model (DPBM) [11] was proposed to predict the bone metastasis of breast cancer patients. In addition, two other classifiers, support vector machine (SVM) and shrunken centroids classifier (SCC), using the same genes in DPBM as features, were also compared with the DPBM in the original work. As our work used the same data sets as the DPBM, we compared our centroid classifier with the DPBM, SVM, and SCC to validate the performance of our work.

**Figure 3:**
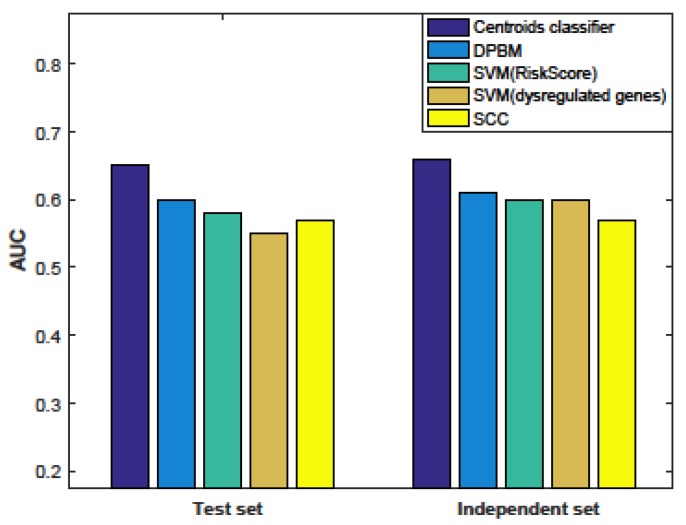
Comparing the performance of our method with published methods. AUC: area under the curve; DPBM: dysregulated pathway-based prediction model. SVM: Support vector machine; SCC: Shrunken centroids classifier.

The AUC (area under the curve) of the four classifiers in the test data set and independent data set are shown in Figure 3 (the details of the performance are shown in Table S3). As a result, our centroid classifier could achieve AUCs of 0.65 and 0.66 in the two data sets, respectively, while the best AUC of all the other methods was about 0.60. That is, our centroid classifier outperformed other methods and our method dramatically improved the predictive performance in bone metastasis.

### 3.6. Evaluation the Significance of Our Method

**Figure 4:**
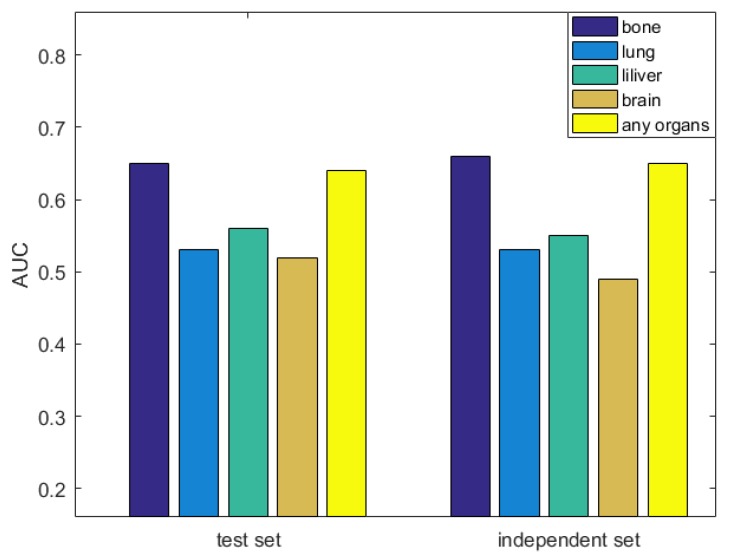
Comparing the performance of bone metastasis with other organ metastases.

We made predictions based on our signature genes in other organ metastases (lung, liver, brain, and any organs) to verify the specificity of bone metastasis. We calculated the AUC and accuracy of five organs in the training, test, and independent data sets. The results are shown in Table S4 and Figure 4. We found that our signature had very limited predictive ability for each of the other organs. In addition, the performance of metastasis prediction in any organs (the traditional overall survival or distant-metastasis-free survival) was good, but slightly worse than bone-metastasis prediction. This is because most of the organ metastases are bone metastases. This illustrates that our signature genes have specificity for bone metastasis. Moreover, it has been reported that the status of a single gene (e.g., estrogen receptor (ER)) can distinguish the bone-metastasis risk in breast cancer [50], so we also compared the performance of ER status and our method in evaluating the risk of bone metastasis of breast cancer. Since ER status listed in the data set is not a continuous risk score but a discrete status, we used the MCC (Matthews correlation coefficient), which is a classification evaluation index for unbalanced data sets, to evaluate the performance of our signature and ER status (Table 3). The results show that our method was better than the use of single genes (e.g., ER status) in classification. Therefore, our gene signature performed better than a traditional clinical index (i.e., ER status) and had specificity for bone metastasis.

## 4. Discussion

Predicting the bone metastasis of breast cancer patients is still a challenge. In this work, we proposed a new method based on the minimal driver gene set in a gene dependency network to predict the risk of bone metastasis of breast cancer.

First of all, we used a merged data set which contained the transcriptome data and clinical information of 855 breast cancer patients. Then, the conditional mutual information was applied to infer the gene dependency pairs in the network for which the mutual information between one gene and the bone-metastasis risk was significantly dependent on the other genes. All the significant gene pairs were constituted as a gene dependency network, and the network was scale free. Besides, case study and function annotation confirmed that the network could reveal the gene dependency relation in the process of bone metastasis. Structure controllability theory states that a system (e.g., biological network) can be controlled by controlling the nodes in the minimal driver gene set. Therefore, the driver nodes in the gene dependency network may be biomarker candidates. However, the size of the minimal driver gene set for the driver sets is too large (In our work, 2483 genes were in each minimal driver gene set). In addition, the solutions of the maximum matching are not unique, especially in complex networks. To solve these problems, we calculated multiple minimal driver gene sets and selected the genes which were involved in all the minimal driver gene sets as feature candidates. Function annotation illustrated that these genes were related to bone metastasis in breast cancer. Based on the feature candidates, 51 genes which were differentially expressed were selected as features, and a centroid classifier was constructed. Our method was validated by survival analysis and through comparison with other methods, and the results showed that our method dramatically improved the predictive performance in bone metastasis.

In conclusion, we provided a new method to study the bone metastasis in breast cancer. The method uncovered the regulation mechanism in bone metastasis by constructing a gene dependency network. A new feature selection method based on the minimal driver gene set in structure controllability theory was proposed to select the key genes. Finally, the prediction model was constructed by using the essential genes as features. In fact, our method can also be used to study other biological problems, as long as there are enough genotypic and phenotypic data. We will validate our method in other biological problems in our future work.

## Figures and Tables

**Table 1:** Functional annotation of the driver gene set.

KEGG Gene Set Name	*p*-Value	FDR q-Value
MAPK signaling pathway	7.31 × 10^−19^	1.36 × 10^−16^
Neuroactive ligand-receptor interaction	1.12 × 10^−15^	1.04 × 10^−13^
Pathways in cancer	2.82 × 10^−13^	1.75 × 10^−11^
Focal adhesion	4.05 × 10^−11^	1.88 × 10^−9^
Cytokine-cytokine receptor interaction	8.22 × 10^−11^	3.06 × 10^−9^
Regulation of actin cytoskeleton	2.72 × 10^−10^	8.43 × 10^−9^
SNARE (SNAP Receptor) interactions in vesicular transport	1.62 × 10^−9^	4.29 × 10^−8^
Complement and coagulation cascades	1.26 × 10^−8^	2.92 × 10^−7^
Purine metabolism	3.08 × 10^−8^	6.36 × 10^−7^
Spliceosome	5.07 × 10^−8^	9.42 × 10^−7^

KEGG: Kyoto Encyclopedia of Genes and Genomes; FDR: False discovery rate.

**Table 2:** The signature genes in our work.

Gene Id	Gene Name	Frequency	*p*-Value
85458	DIXDC1	500	1.68192 × 10^−6^
29068	ZBTB44	500	3.29096 × 10^−6^
51232	CRIM1	500	1.46030e × 10^−5^
9986	RCE1	500	2.12898 × 10^−5^
56888	KCMF1	500	4.00030 × 10^−5^
1456	CSNK1G3	500	4.58237 × 10^−5^
6256	RXRA	500	7.63144 × 10^−5^
55343	SLC35C1	500	0.00012
55520	ELAC1	500	0.00012
55081	IFT57	500	0.00012
57610	RANBP10	500	0.00018
5877	RABIF	500	0.00018
25839	COG4	500	0.00019
23261	CAMTA1	500	0.00020
3009	HIST1H1B	500	0.00020
3092	HIP1	500	0.00026
246243	RNASEH1	500	0.00030
3104	ZBTB48	500	0.00031
10342	TFG	500	0.00032
6282	S100A11	500	0.00033
10462	CLEC10A	500	0.00033
51199	NIN	500	0.00041
10531	PITRM1	500	0.00048
9856	KIAA0319	500	0.00049
11167	FSTL1	500	0.00049
3993	LLGL2	500	0.00052
56729	RETN	500	0.00054
51514	DTL	500	0.00054
9202	ZMYM4	500	0.00058
51302	CYP39A1	500	0.00065
9971	NR1H4	500	0.00067
79083	MLPH	500	0.00073
65082	VPS33A	500	0.00075
10179	RBM7	500	0.00078
55794	DDX28	500	0.00082
57405	SPC25	500	0.00089
51659	GINS2	500	0.00089
1852	DUSP9	500	0.00092
57017	COQ9	500	0.00096
10397	NDRG1	500	0.00098
9911	TMCC2	500	0.00128
55095	SAMD4B	500	0.00137
23649	POLA2	500	0.00143
10615	SPAG5	500	0.00143
7134	TNNC1	500	0.00145
7083	TK1	500	0.00146
9442	MED27	500	0.00151
8449	DHX16	500	0.00171
8817	FGF18	500	0.00176
483	ATP1B3	500	0.00179
2175	FANCA	500	0.00190

The frequency of a gene is the times involved in the minimal driver gene sets and the *p*-values were calculated based on *t*-test.

**Table 3:** Comparing results with estrogen receptor (ER) status.

	Training Data Set	Test Data Set	Independent Data Set
Centroids classifier	0.22	0.15	0.20
ER status	0.18	0.16	0.07

## Supplementary Materials

The following are available online at https://www.mdpi.com/2073-4425/10/6/466/s1, Figure S1: The gene dependency network, Table S1: The gene dependency network of bone metastasis in breast cancer, Table S2: The feature candidates, Table S3: Comparing results with other methods, Table S4: Comparing results with other organs.
