# Supplementary material for: Prediction of Bone Metastasis in Breast Cancer Based on Minimal Driver Gene Set in Gene Dependency Network
Source: Genes (Basel). 2019 Jun 17;10(6):466. doi: 10.3390/genes10060466 (PMC6627827; doi:10.3390/genes10060466)
Supplement: Supplementary file 1 [file genes-10-00466-s001.zip › genes-482569-s-proof.docx]

Supplementary Material: Prediction of bone metastasis in breast cancer based on minimal driver gene set in gene dependency network

**1** **Supplementary Tables**

There are four tables in the supplementary material. Table S1 (The gene dependency network of bone metastasis in breast cancer) and Table S2 (The feature candidates) are in separate documents.

Table S3. Comparing our results with other methods.

|  | Training data set | | Test data set | | Independent data set | |
| --- | --- | --- | --- | --- | --- | --- |
|  | AUC | Accuracy | AUC | Accuracy | AUC | Accuracy |
| Centroids classifier | 0.62 | 0.62 | 0.65 | 0.62 | 0.66 | 0.54 |
| DPBM | 0.76 | 0.64 | 0.60 | 0.60 | 0.61 | 0.66 |
| SVM (RiskScore) | 0.72 | 0.71 | 0.58 | 0.59 | 0.6 | 0.6 |
| SVM (dysregulated genes) | 0.75 | 0.75 | 0.55 | 0.54 | 0.60 | 0.59 |
| SCC | 0.78 | 0.65 | 0.57 | 0.55 | 0.57 | 0.44 |

This table describes the AUC and the accuracy of our work and other methods in training, test, and independent data sets.

Table S4. Comparing results with other organs.

|  | Training data set | | Test data set | | Independent data set | |
| --- | --- | --- | --- | --- | --- | --- |
|  | AUC | Accuracy | AUC | Accuracy | AUC | Accuracy |
| bone | 0.62 | 0.62 | 0.65 | 0.62 | 0.66 | 0.54 |
| lung | 0.58 | 0.56 | 0.53 | 0.48 | 0.53 | 0.51 |
| liver | 0.60 | 0.59 | 0.56 | 0.57 | 0.55 | 0.46 |
| brain | 0.55 | 0.56 | 0.52 | 0.55 | 0.49 | 0.58 |
| any organs | 0.62 | 0.62 | 0.64 | 0.66 | 0.65 | 0.57 |

This table describes the AUC and accuracy of bone metastasis and other organs in the training, test, and independent data sets.

**2 Supplementary Figure**

There is one figure in the supplementary material.


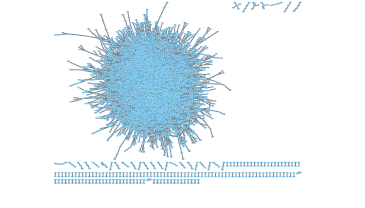


**Figure S1.** The gene dependency network. In this figure, the edge from node B to node A means the correlation between B and the phenotype is significantly dependent on gene *A*.
